# Anxiety and Depression Symptoms among Youth Survivors of Childhood Sexual Abuse: A Network Analysis

**DOI:** 10.1186/s40359-023-01275-3

**Published:** 2023-09-16

**Authors:** Jiaqi Li, Yu Jin, Shicun Xu, Xianyu Luo, Amanda Wilson, Hui Li, Xiaofeng Wang, Xi Sun, Yuanyuan Wang

**Affiliations:** 1 Key Laboratory of Brain, Cognition and Education Sciences, Ministry of Education, China; School of Psychology, Center for Studies of Psychological Application, and Guangdong Key Laboratory of Mental Health and Cognitive Science, South China Normal University, Guangzhou, China; 2 College of Education for the Future, Beijing Normal University, Beijing, China; 3 Northeast Asian Research Center, Jilin University, Changchun, China; 4 Department of Population, Resources, and Environment, Northeast Asian Studies College, Jilin University, Changchun, China; 5 China Center for Aging Studies and Social-Economic Development, Jilin University, Changchun, China; 6 Division of Psychology, Faculty of Health and Life Sciences, De Montfort University, Leicester, UK; 7 School of Public Health, Jilin University, Changchun, China

**Keywords:** Childhood sexual abuse, Young adults, Comorbidity, Anxiety, Depression, Network analysis

## Abstract

**Background:**

Previous studies have frequently reported a high prevalence of co-occurring anxiety and depression among people who experienced stressful events in childhood. However, few have noted the symptomatic relationship of this comorbidity among childhood sexual abuse (CSA) survivors. Therefore, this study’s objectives were as follows: (1) to examine the relationship across symptoms between anxiety and depression among CSA survivors; (2) to compare differences between male and female network structures among CSA survivors.

**Methods:**

A total of 63 Universities and Colleges in Jilin Province, China, covered 96,218 participants in this study, a sub-set data of which met the criteria of CSA was analyzed with the network analysis. The Childhood Trauma Questionnaire-Short Form (CTQ-SF), measured CSA. Anxiety was measured by the seven-item Generalized Anxiety Disorder Scale (GAD-7), and depression was measured by the Patient Health Questionnaire (PHQ-9). The sex difference between anxiety and depression among CSA survivors was compared.

**Results:**

3,479 college students reported the experience of CSA (CTQ-SF total scores ≥ 8), with a prevalence of 3.62% (95% CI: 3.50–3.73%). Among CSA survivors, control worry, sad mood, and energy were central and bridge symptoms of the anxiety and depression network. Meanwhile, male CSA survivors appeared to have a stronger correlation between guilt and suicide, but female CSA survivors seemed to have a stronger correlation between control worry and suicide. Moreover, the edge of control worry-relax-afraid was stronger in the male network, while the edge of restless-relax was stronger in the female network.

**Conclusion:**

Control worry, sad mood, and energy are crucial to offer targeted treatment and to relieve anxiety and depression symptoms for CSA survivors. Guilt needs more attention for male CSA survivors, while control worry remains more important for female CSA survivors to reduce suicidal ideation and suicide attempts.

**Supplementary Information:**

The online version contains supplementary material available at 10.1186/s40359-023-01275-3.

## Introduction

Over the past several decades, the prevalence of childhood sexual abuse (CSA) has increased, ranging from 4 to 20% across the world [1], making it a global public mental health concern. In China, the overall prevalence of CSA ranges from 8.9 to 27.5% [2–4]. The World Health Organization defined CSA as the sexual involvement inflicted upon a child who is under the age of 18 years who may not fully comprehend sex, has the inability or un-prepared development to give informed consent, or is an act in violation of the social laws and taboos [5]. Literature suggests that experiences of CSA could permanently harm mental and physical development across the life span [6, 7]. Meanwhile, it is further reported that survivors of CSA suffer from severe damage to their self-esteem [8], which may hinder an individual’s overall well-being [9] and increase risks of suicidal ideation and attempts [10]. Reviews also reported that persons with a history of CSA are potentially at risk of long-term anxiety and depression diagnoses [11, 12]. A meta-analysis included studies based on non-clinical samples found that individuals with a history of CSA may sustainably tolerate various mental disorders, in particular, bearing the burden of anxiety [OR = 1.90 (95% CI: 1.60–2.25)] and depression symptoms [OR = 2.11 (95% CI: 1.83–2.44)] [13]. Another longitudinal cohort study of non-clinical individuals summarized the extent of CSA significantly exerted positive associations with major depression (β = 0.426, SE = 0.094, P < 0.001) and anxiety disorder (β = 0.364, SE = 0.089, P < 0.001) [14].

The commonly known risk consequences of CSA are correlated to psychiatric or psychopathological problems [15, 16]. According to the cognitive perspective, CSA, as one of the stressful events in childhood, enables the formation of maladaptive schemas for a person [17]. Such a complex stressor could disrupt crucial cognitive functions such as emotional regulation, negatively reinforcing an individual’s psychiatric problems [18]. As the ecological system theory states, children are in a rapid developmental period of physical and mental health, mainly influenced by the microsystem of family members, and mesosystem like the interaction between parents and school [19]. However, due to being sexually abused in childhood, adolescent development would be impaired, exerting constant negative influence throughout an individual’s adult life. In particular, good cognitive performance appears more important for college students, and adverse childhood experiences hinder functioning due to numerous biological systems, such as reduced hippocampal volume, which would affect college students’ academic achievement [20]. Psychologically, young adults may fail to build stable self-consciousness and self-esteem after being sexually abused as a child [21]. With the gradual maturation of the cognitive system, rumination of past CSA could trigger retrospective traumatic memories, increasing risks to mental health as well [22]. All these stressors would aggravate the individuals’ risks of anxiety, depression, and even suicidal behaviors [23]. Therefore, considering the brain development of young college students, it is essential to investigate the constant impact of CSA on their mental health.

Previous research has continuously reported that compared with non-abused young adults, survivors of CSA have a higher possibility of co-occurring anxiety and depression [24, 25]. An umbrella review reported that anxiety [OR = 2.7 (95% CI: 2.5–2.8)], and depression [OR = 2.7 (95% CI: 2.4–3.0)] frequently occurred one after another among adults over 18 years with experiencing sexual abuse as a child [11]. Theoretical and empirical works have attempted to explain this comorbid phenomenon, with a two-factor model accounting for the comorbidity of various psychiatric disorders, indicating that a general psychopathological factor (P factor) summarizes every form of the psychiatric problem resulting from having a poor developmental history [26]. A possible neurobiological trajectory framework of pathogenesis suggests that the co-occurring psychiatric disorder pathway might be an aggregation of genetic, biological, and psychosocial factors [15]. Once subjected to CSA, neurological systems connecting to emotional functions could be a deficit, such as decreased thickness in the ventromedial prefrontal cortex, damage to neurons in the hippocampus, and injury of the prefrontal cortex and amygdala [27]. Compared with non-abused adults, individuals who experience CSA were found to have primarily reduced hippocampal volume as well [28]. All the above factors could help to explain the reasons for CSA survivors presenting with more severe anxiety and depression symptoms.

In addition, research have found sex differences, with several studies suggesting differences in mental outcomes even among male and female youth who experienced CSA. Women with a history of CSA have stronger anxiety and depression symptoms than men [29]. This may relate to the difference in the male and female neural functioning structures, in which male adults feel fatigued more from depression and female adults from anxiety [30]. Moreover, comorbid anxiety and depression result in a two-fold higher risk of suicide in men than in women [31]. However, studies are scarce exploring the male and female distinctions in the symptomatic relationship between anxiety and depression among adult survivors of CSA, which acts as our study’s investigation point.

The network approach, a relatively novel research method to explore various relationships across symptoms, has been widely applied in psychopathological and clinical psychiatric fields [32, 33]. As a systematic review noted, network analysis has been applied to the general or clinical samples to investigate a plethora of psychiatric disorders, including anxiety and depression in adolescents and adults [34]. For example, a network analysis on anxiety and depression among non-clinical undergraduate students found that “sad mood” had a high central and bridge influence in the comorbidity network structure of these two disorders [35]. In a network, each symptom is regarded as a node, and each edge is viewed as a possible relationship between any two nodes, whose weight is defined by its partial correlation coefficient [36]. The expected influence (EI) index indicates the centrality of each symptom, and via it, the most central symptoms could be identified in a network [37]. Moreover, bridge symptoms, connecting and activating each other through their linked symptoms, exert influence from one disorder to another, which can be estimated by bridge expected influence (bEI) indices [38]. Both central and bridge symptoms play a role in relieving symptoms of various psychiatric disorders. Based on these characteristics, the network analysis method provides an innovative way to explore the crucial role of a specific symptom, or a connection of symptoms, in a given disorder. In addition, it can identify mutual dynamics among symptoms of given disorders more so than purely general relationships across various disorders [34]. However, few previous studies considered a network approach to investigate how symptoms of anxiety and depression present in young adults who have experienced CSA. A scarcity of published studies explored the associations of symptoms between anxiety and depression among young adults with CSA. Therefore, this study aimed to reveal the potential symptom network structures of anxiety and depression among adult CSA survivors and propose possible measures to relieve their symptoms.

## Methods

### Study design and settings

This cross-sectional study investigated mental health among adults who experienced CSA. Following the Strengthening the Reporting of Observational Studies in Epidemiology (STROBE) guidelines [39], the data was collected online in Jilin Province, China, from October to November 2021. Utilizing a Quick Response code, students in 63 Universities and Colleges completed a questionnaire with informed consent. Inclusion criteria included: (1) only 16 or older young adults; (2) students who study in Colleges or Universities in Jilin Province, China; (3) able to understand Chinese and the assessment materials. Participants were excluded from this study for several reasons: (1) failed to answer at least three in four questions on attention detection correctly; (2) abnormal and illogical answers on age and other questions of blank filling.

Jilin University granted this study ethical approval according to the 1964 Helsinki Declaration and its amendments in 2013, and with ethical standards. All participants provided electronic informed consent.

### Measurements

#### Childhood sexual abuse

The Chinese version of the Childhood Trauma Questionnaire-Short Form (CTQ-SF) was utilized to screen the experience of CSA [40], developed by the version of Bernstein and colleagues [41]. It is a retrospective self-report scale that includes 28 items to assess childhood trauma experiences, divided and collected into five subtypes: emotional abuse, physical abuse, sexual abuse, emotional neglect, and physical neglect. Participants answered each item using a 5-point Likert scale ranging from 1 (“never true”) to 5 (“very often true”). The total scores are summed based on the self-report Likert scale in relation to each question. Different optimal cut-off scores are recommended to screen for positive participants in each of the five subscales [42]. The cut-off score for emotional abuse (EA) is ≥ 13, which is considered moderate-severe EA. The cut-off score for physical abuse (PA) is ≥ 10, and classified as moderate-severe PA. For sexual abuse, the cut-off score is ≥ 8, inclusive of moderate to severe exposure (CSA in this study). In addition, the cut-off score is ≥ 10 in the physical neglect subscale, and ≥ 15 in the emotional neglect subscale. In order to avoid the interaction between each form of abuse, participants who met the criteria for more than one form of abuse were excluded from the analyzed sample. Finally, a total of 3,479 participants suffered CSA. The Chinese version of CTQ-SF has a Cronbach’s alpha of 0.79, showing acceptable reliability among the undergraduate sample [43]. In addition, the CTQ-SF shows a good reliability of 0.854 in this total sample.

#### Anxiety symptoms

The Chinese version of the seven-item Generalized Anxiety Disorder Scale (GAD-7) was used to measure the severity of anxiety [44]. The GAD-7 consists of seven items: (1) “Nervous”; (2) “Control Worry”; (3) “Worry A Lot”; (4) “Relax”; (5) “Restless”; (6) “Irritable”; (7) “Afraid”. The GAD-7 scale has a good test-retest reliability of 0.856, with a sensitivity of 86.2% and a specificity of 95.5%, using 10 as the cutoff [45]. In addition, the GAD-7 shows good reliability of 0.912 in this sample.

#### Depression symptoms

The Chinese version of the nine-item Patient Health Questionnaire (PHQ-9) was utilized to assess the severity of depression [46]. The PHQ-9 consists of nine items: (1) “Anhedonia”; (2) “Sad Mood”; (3) “Sleep”; (4) “Energy”; (5) “Appetite”; (6) “Guilt”; (7) “Concentration”; (8) “Motor”; (9) “Suicide”. Previous studies indicate that the PHQ-9 scale could be a valid and reliable measurement among the general population [47]. The PHQ-9 also performed well in the Chinese population, with an internal consistency reliability of 0.86. A cut-off score of 7 or higher on the PHQ-9 had a sensitivity and specificity of both 86% [48]. In addition, the PHQ-9 shows good reliability of 0.879 in this sample.

### Statistical analysis

#### Descriptive analysis

This study retained 96,218 participants after excluding the inefficient data from a larger data set of 117,769. The remaining participants were extracted to form the sub-data (n = 3,479) who met the cut-off for CSA (CTQ-SF total scores ≥ 8) and were analyzed with R programming software and IBM SPSS version 26.0.

All University students in the sub-set data set were divided into two groups according to their birth sex (male or female). Sociodemographic variables included participants’ age, residence, ethnicity, family type, current annual income, and whether they have siblings. Age and scores of the measures (i.e., GAD-7, PHQ-9) were continuous variables; all the other sociodemographic variables were considered categorical and classified into two or more types. The categorical variables were compared using Chi-square analysis, and continuous variables were compared using independent-sample t-tests to investigate the differences in the male and female groups.

#### Network estimation

Statistics analysis was conducted with R statistical programming language [49]. The mean difference (MD), standard deviation (SD), and predictability of the GAD-7 and the PHQ-9 items were computed, respectively. Based on network analysis theory, a “node” is used to represent a symptom in a network, and an “edge” is used to describe the correlation between any two nodes [32]. Estimating the Graphical Gaussian Model (GGM) represent the structure of anxiety and depression in terms of the severity of symptoms [50]. The associations of edges are the partial correlation coefficient between two nodes after controlling all other variable influences in the network. And the thicker the non-directed node between two edges, the higher the partial correlation coefficient between them. Using the graphic least absolute shrinkage and selection operator (LASSO) [51], the network was regulated to avoid overfitting and to elevate the interpretability of a network model structure. Meanwhile, the shrinkage parameter could be selected based on the Extended Bayesian Information Criterion (EBIC) [52]. The centrality of each node was identified according to the expected influence (EI), which represents the whole edges of the weight connecting to this code [53]. The bridge expected influence (bEI) was used to measure the linkage between two nodes in anxiety and depression symptoms. The ‘qgraph’ R package [54] was employed to visualize the symptomatic correlation of a network. Considering several sociodemographic variables existed significant differences between male and female participants on CSA experiences, to further investigate whether confounding variables would affect the estimated model, an adjusted network model was re-estimated after controlling sex, family type, current annual income, and only-child status. Finally, Spearman’s rank correlation and an independent-sample t-test were utilized to compare the difference between the original and adjusted network structures.

#### Network comparison

Network models of male and female participants were compared utilizing the Network Comparison Test (NCT) [53], a permutation hypothesis test conducted with the R package ‘NetworkComparisonTest’. At first, the strength of the global network was estimated on a sex-specific subsample, utilizing 1,000 permutations by comparing absolute values of each edge weight among the network. Since each edge weight was compared, we used multiple tests proposed by Holm to correct P values. Secondly, by subtracting the edge values of the female network from counterparts of the male network, the differences between every two edges were calculated, which means the differences of correlations of each two nodes. Finally, an independent t-test was applied to examine the significant differences between male and female participants.

#### Network stability

It is recommended to examine the accuracy and stability of the network after estimation with the bootstrapping method [55]. At first, we calculated the stability of the edge weights by applying a non-parametric bootstrapping method. After this step, 95% of generated confidence intervals (CIs) were calculated again in a random re-sampled dataset. Second, we calculated the correlation stability co-efficiency (CS-C), which defines centrality stability. CS-C represents the maximum portions that could be excluded to obtain a probability of 95%, which makes the ranking correlation between the original centrality indices and case-subset indices reach a significant effect at 0.7. Thus, the centrality indices should be recommended to be interpreted with a CS-S above 0.25 and preferentially above 0.5. Finally, 95% nonparametric bootstrap CIs of differences between each pair of edges weight or node indices were used to identify the significant difference between them.

## Results

### Descriptive statistics

Table 1 presents the socio-demographic characteristics of participants. Among the 96,218 participants, 3.62% (95% CI: 3.50–3.73%) reported experiencing CSA (n = 3,479). The CSA sample consisted of young adults above 16 years (M = 19.67, SD = 4.05) with an even distribution of sex (48.83% female, 51.16% male). The prevalence of CSA among males was 4.2% (95% CI: 4.04–4.44%), while among females was 3.2% (95% CI: 3.03–3.32%). In addition, the prevalence of CSA showed significant sex differences in items of family composition (χ²=29.14, P < 0.001), with most being nuclear family type (65.4%); current annual income (χ²=34.45, P < 0.001), with most income being under $2169 (60.4%); and whether they were only-child status (χ²=84.87, P < 0.001), with most having siblings (51.2%). There were also significant differences in scores of GAD-7 (t=-7.04, P < 0.001) and PHQ.9 (t=-6.73, P < 0.001) between the male and female groups. The mean difference, standard deviation, and average predictability of each symptom are reported in Table 2.

**Table 1:** Socio-demographic characteristics of participants with a history of childhood sexual abuse

	Sexual abuse	Male	Female	Х²	P value
	(N = 3479)	(N = 1699)	(N = 1780)		
Residence					
City	1788 (51.4)	850 (50.0)	938 (52.7)	2.48	0.116
Town and county	1691 (48.6)	849 (50.0)	842 (47.3)		
Ethnicity					
Han	3119 (89.7)	1548 (91.1)	1571 (88.3)	7.63	0.006
Others	360 (10.4)	151 (8.9)	209 (11.7)		
Family type					
Nuclear family	2272 (65.4)	1124 (66.2)	1148 (64.5)	29.14	**< 0.001**
More than three generations	651 (18.7)	356 (21)	295 (16.6)		
Other	556 (16.0)	219 (12.9)	337 (18.9)		
Current annual income					
<$930	1052 (30.3)	498 (28.0)	554 (31.1)	34.45	**< 0.001**
$930-$2169	1047 (30.1)	464 (26.1)	583 (32.8)		
$2170–3565	553 (15.9)	262 (14.7)	291 (16.3)		
>3565	827 (23.8)	475 (26.7)	352 (19.8)		
Only-child					
Yes	1698 (48.8)	965 (56.8)	733 (41.2)	84.87	**< 0.001**
No	1781 (51.2)	734 (43.2)	1047 (58.8)		
				T	
Age, years: Mean (SD)	19.67 (4.05)	19.60 (1.74)	19.53 (1.74)	1.214	0.225
GAD-7	5.18 (4.34)	4.65 (4.33)	5.68 (4.29)	-6.729	**< 0.001**
PHQ-9	6.91 (4.92)	6.34 (4.98)	7.45 (4.80)	-7.040	**< 0.001**

Note: GAD-7, the seven-item Generalized Anxiety Disorder Scale (GAD-7); PHQ-9, the nine-item Patient Health Questionnaire; Х², the statistics of Chi-square tests; T, the statistics of T-tests; P value, the bold one refers to significant differences between groups; 1 US dollar = 6.4512 RMB yuan.

**Table 2:** Basic information of scales and descriptive item statistics

Scale	Symptoms	Items	Mean (SD)	Predictability
GAD-7	Nervous	1.Feeling nervous, anxious or on edge	0.86(0.73)	59%
	Control Worry	2.Not being able to stop or control worrying	0.79(0.79)	66%
	Worry A Lot	3.Worrying too much about different things	0.89(0.82)	63%
	Relax	4.Trouble relaxing	0.81(0.82)	60%
	Restless	5.Being so restless that it is hard to sit still	0.53(0.70)	50%
	Irritable	6.Becoming easily annoyed or irritable	0.76(0.77)	57%
	Afraid	7.Feeling afraid as if something awful might happen	0.54(0.71)	49%
PHQ-9	Anhedonia	1.Little interest or pleasure in doing things	0.87(0.73)	43%
	Sad Mood	2.Feeling down, depressed or hopeless	0.83(0.69)	54%
	Sleep	3.Trouble falling asleep, staying asleep, or sleeping too much	0.89(0.86)	41%
	Energy	4.Feeling tired or having little energy	1.01(0.80)	56%
	Appetite	5.Poor appetite or overeating	0.85(0.84)	37%
	Guilt	6.Feeling bad about yourself – or that you’re a failure or have let yourself or your family down	0.77(0.79)	47%
	Concentration	7.Trouble concentrating on things, such as reading the newspaper or watching television	0.88(0.85)	47%
	Motor	8.Moving or speaking so slowly that other people could have noticed;or, the opposite – being so fidgety or restless that you have been moving around a lot more than usual	0.53(0.72)	44%
	Suicide	9.Thoughts that you would be better off dead or of hurting yourself in some way	0.29(0.57)	31%

Note: SD, standard deviation; GAD-7, the seven-item Generalized Anxiety Disorder Scale; PHQ-9, the nine-item Patient Health Questionnaire.

### Network structure

**Fig. 1:**
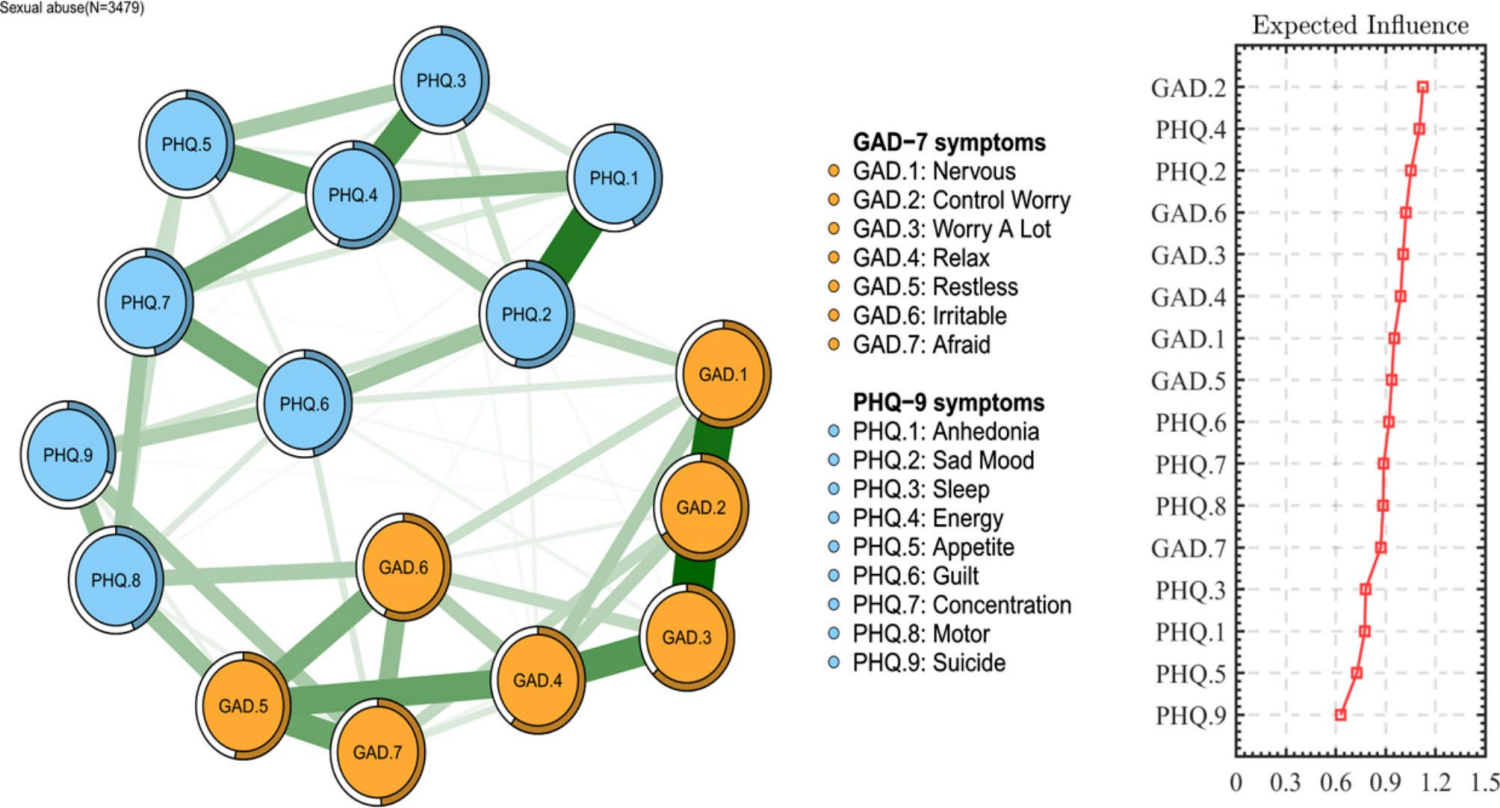
The network structure of anxiety and depression among CSA survivors

**Fig. 2:**
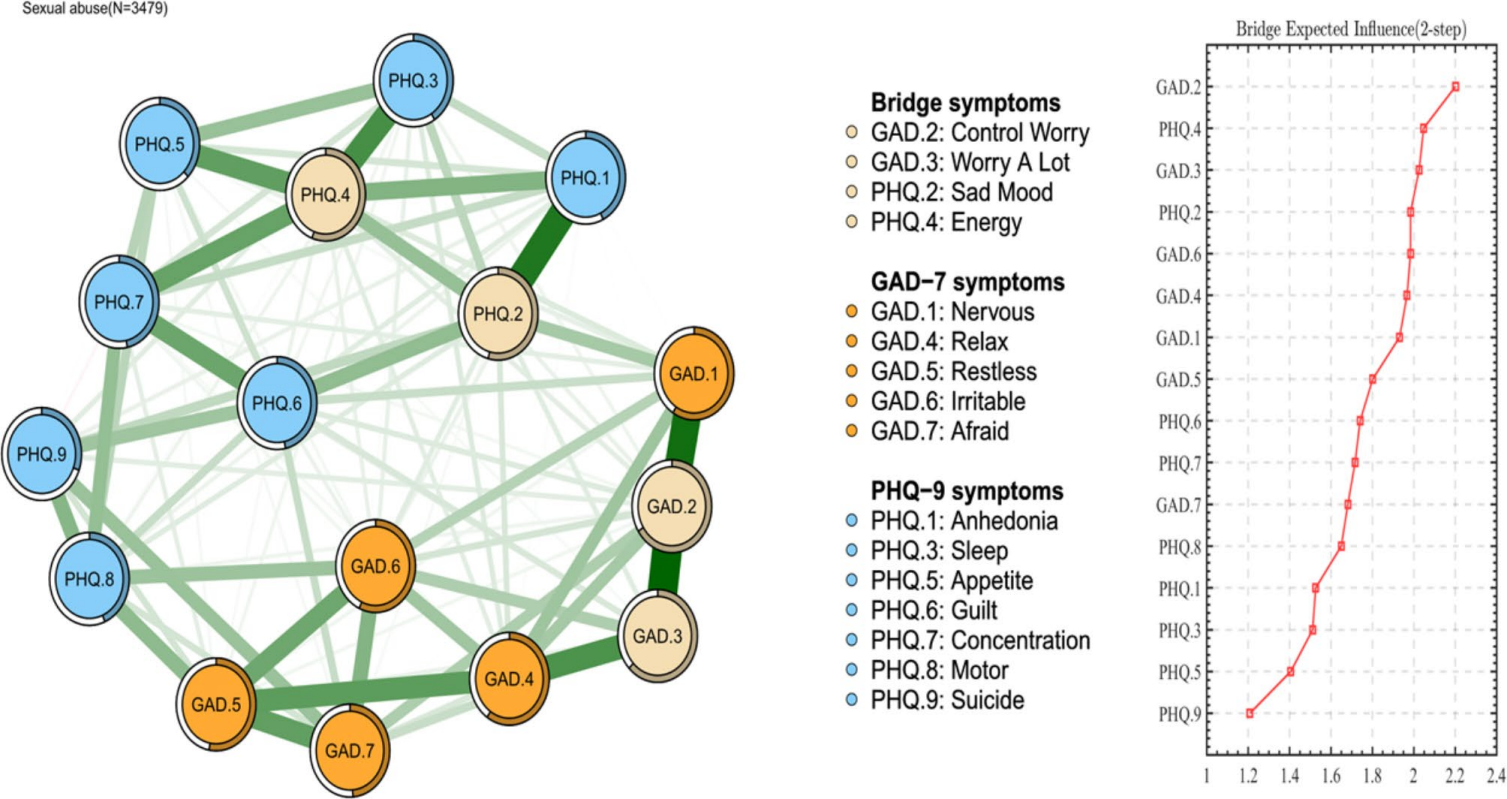
Network structure of bridge symptoms of anxiety and depression among CSA survivors

Figure 1 shows the global network structure between the GAD-7 and PHQ-9 symptoms. Figure 2 shows the network structure of bridge symptoms of anxiety and depression among adults who experienced CSA. The average predictability of nodes was 50%, which means that, on average, the variance of 50% per node could be explained by its neighboring nodes. After controlling confounding variables, including sex, family type, current annual income, and only-child status, the adjusted global network structure was significantly correlated with the original one (r = 0.76, P < 0.001) (Figure S1). The t-test result suggested that the confounding variables did not significantly affect the network model (t = 0.89, P = 0.38).

In terms of centrality index EI, node GAD.2 (“Control Worry”) had the highest EI centrality in the network, followed by node PHQ.4 (“Energy”), node PHQ.2 (“Sad Mood”) and GAD.6 (“Irritable”). As shown in Fig. 1, it indicates that these four symptoms have the most severe reports and the most meaningful impact on the structure of the network model for clinicians to understand anxiety and depression among adults who have experienced CSA. (Figure S2) In the aspect of bEI, GAD.2 (“Control Worry”), PHQ.4 (“Energy”), GAD.3 (“Worry A Lot”), and PHQ.2 (“Sad Mood”) were the most critical bridge symptoms connecting anxiety and depression. (As shown in Fig. 2, Figure S3)

Figure 1 also showed the relationship of the symptoms network revealed that in the anxiety symptoms, the strongest association was between nodes GAD.2 (“Control Worry”) and GAD.3 (“Worry A Lot”), followed by nodes GAD.1 (“Nervous”) and GAD.2 (“Control Worry”), nodes GAD.3 (“Worry A Lot”) and GAD.4 (“Relax”), and nodes GAD.4 (“Relax”) and GAD.5 (“Restless”). In the depression symptoms, the strongest correlation was between nodes PHQ.1 (“Anhedonia”) and PHQ.2 (“Sad Mood”), followed by nodes PHQ.3 (“Sleep”) and PHQ.4 (“Energy”), nodes PHQ.4 (“Energy”) and PHQ.5 (“Appetite”), and nodes PHQ.4 (“Energy”) and PHQ.7 (“Concentration”). The network structure of anxiety and depression showed good stability, among CSA victims, the connection between GAD.5 and PHQ.8 (mean edge weight = 0.14) was the strongest edge, followed by the relationship between GAD.7 and PHQ.9 (mean edge weight = 0.13) and the relationship between GAD.6 and PHQ.8 (mean edge weight = 0.13). (Figure S4, Figure S5, Figure S6)

### Network comparison tests for sex

**Fig. 3:**
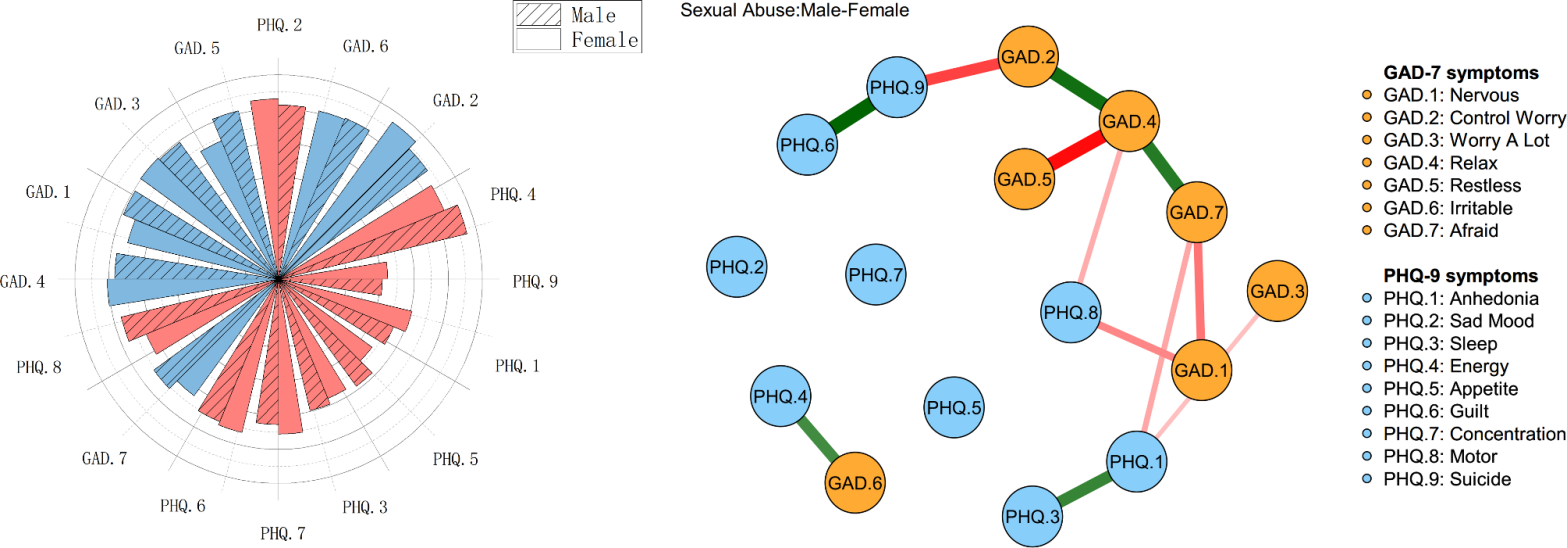
Network comparison of male and female participants among CSA survivors

Comparing the network models between male and female participants, different interactions within the two networks were observed in this study. As shown in Fig. 3, according to the green edges of node PHQ.6 (“Guilt”) and PHQ.9 (“Suicide”), node GAD.2 (“Control Worry”) and GAD.4 (“Relax”), node GAD.4 (“Relax”) and GAD.7 (“Afraid”), PHQ.1 (“Anhedonia”) and PHQ.3 (“Sleep”), node PHQ.4 (“Energy”) and GAD.6 (“Irritable”), and connections of these edges were closer among male CSA survivors than those of their female counterparts. Whereas, according to the red edges of node GAD.2 (“Control Worry”) and PHQ.9 (“Suicide”), node GAD.4 (“Relax”) and GAD.5 (“Restless”), node GAD.1 (“Nervous”) and GAD.7 (“Afraid”), node GAD.1 (“Nervous”) and PHQ.8 (“Motor”), GAD.4 (“Relax”) and PHQ.8 (“Motor”), GAD.7 (“Afraid”) and PHQ.1 (“Anhedonia”), and GAD.3 (“Worry A Lot”) and PHQ.1 (“Anhedonia”), female CSA survivors had closer connections on these edges than male CSA survivors.

Both in the male network and the female network, the strongest correlation was the edge between node GAD.2 (“Control Worry”) and GAD.3 (“Worry A Lot”), the most central symptoms were node GAD.2 (“Control Worry”) and PHQ.4 (“Energy”), the most crucial bridge symptom was node GAD.2 (“Control Worry”). Meanwhile, in the male network, GAD.6 (“Irritable”) and PHQ.4 (“Energy”) were both central and bridge symptoms, while in the female network, PHQ.2 (“Sad Mood”) was both a central and bridge symptom. (Figures S7, Figure S8, Figure S9, Figure S10)

In addition, statistically significant differences in the male and female network structures were demonstrated in this study. (Table S1) In the anxiety network, sex appeared significantly different on nodes GAD.1 (“Nervous”), GAD.2 (“Control Worry”), and GAD.3 (“Worry A Lot”). In the depression network, sex showed significant differences on PHQ.1 (“Anhedonia”), PHQ.2 (“Sad Mood”), and PHQ.9 (“Suicide”). The stability of the male and female networks are shown in Figure S11. Male and female networks’ results (using bootstrapping, 95% CI for edges and bootstrapped differences tests for edge weight) are presented in Figure S12. The results of the estimation of edge weight difference by bootstrapped difference test are pictured in Figure S13.

## Discussion

In this study, for the first time, the network approach was conducted to analyze the relationship of symptoms between anxiety and depression among survivors of CSA. “Control worry”, “Sad mood”, and “Energy” were both central and bridge symptoms, which indicated these three symptoms were the most severe in the comorbid anxiety and depression network of CSA survivors. Meanwhile, male CSA survivors appeared to have a stronger correlation between guilt and suicide, but female CSA victims seemed to have a stronger correlation between controlling their worry and suicide. Furthermore, the edge of control worry-relax-afraid was stronger in the male network, while the edge of restless-relax was stronger in the female network.

The current study found that “Control worry”, “Sad mood”, and “Energy” were identified as the most critical symptoms in the network of anxiety and depression among CSA survivors. These findings parallel the results of earlier network studies on various clinical and non-clinical samples. For example, a previous network analysis conducted on a large international non-clinical sample also found that worry emerged as a crucial symptom in the interaction between anxiety and depression [56]. In two network analyses based on clinical psychiatric samples, sad mood was the most central symptom in the anxiety and depression network structure, followed by uncontrollable worry [37, 57]. Also, a near-term study on non-clinical undergraduate students supported that sad mood had a high centrality and bridge centrality in the anxiety and depression network structure [35]. One study on patients with clinical major depression found that loss of energy or fatigue was the most central symptom in the anxiety and depression network [58], and the same finding was stated in another network analysis based on non-clinical nursing undergraduate students [59].

In this study, “Control worry” refers to being unable to stop or control worry, frequently identified as one central or bridge symptom in the network structure of comorbid anxiety and depression [60, 61]. Worry has been hypothesized to be a core transdiagnostic process crossing current mental disorders diagnostic boundaries [62]. Empirical studies indicate that worry exerts specific effects on cardiac activity [63], and it increases the activity of the sympathetic nervous system while decreasing the activity of the parasympathetic nervous system [64]. Survivors of CSA also appear to have a higher sympathetic nervous system response to sex-related stimuli, [65], which may aggravate worries as well. All these simultaneously result in more co-occurring negative emotions and physical arousal symptoms [66]. Meanwhile, an increased sad mood may generate worries over catastrophizing (thinking something happens with catastrophic steps) [67]. Sad mood, referring to feeling down, depressed, or hopeless, was more central than most other symptoms in the anxiety and depression network [68]. Neuroimaging results identified that sad mood affects the thalamus, a specific brain region of cognition linked to emotional state [69]. Earlier studies have explored that exposure to sexual abuse may affect smaller thalamic volume as well [70, 71]. Thus, those with an experience of CSA may incur more severe sad moods due to the smaller volume. Energy refers to fatigue, feeling tired, or having little energy, which was the central symptom and strongly correlated with other symptoms within the anxiety and depression network [59]. Fatigue symptoms seemed more likely to be predicted by CSA among all childhood trauma subtypes [72], which indicated fatigue was one typical symptom for the studied groups. Chronic fatigue syndrome results in hypercortisolism [73], impairing the adrenal cortex’s responsiveness to adrenocorticotropic hormones [74], and it may be connected to dysfunction of the immune system [75], which has further impacts on mental health. A systematic review showed that the immune system of CSA survivors could be damaged and then inhibits the secretion of cortisol from the adrenal cortex, whose analogical mechanism may exacerbate fatigue [76]. Thus, control worry, sad mood, and lack of energy incur various adverse effects on psychiatric mechanisms, which may aggravate the prevalence of comorbid depression and anxiety among CSA survivors.

However, our results could have been inconsistent with previous studies in some aspects. For instance, within the comorbid anxiety and depression network structure, Beard and colleagues [37] found that sad mood and worry were central symptoms but without mentioning these as being bridge symptoms like in our study. Park and colleagues [58] reported that lack of energy was the most central symptom but not the bridge symptom as in this study. Ren and colleagues [59] found that fatigue was the central symptom, but sad mood was the bridge symptom. The difference may be due to the following points. On the one hand, compared with other populations, CSA survivors may appear to have a specific tendency towards the aforementioned three vital symptoms. For instance, compared with non-abused populations, individuals who experienced CSA were found to have primarily reduced hippocampal volume that influenced emotional regulation [28]. On the other hand, adaptive scales are inconsistent across the mentioned studies, such as the BDI.II scale versus the PHQ-9 scale, which may account for the different results.

Moreover, male survivors of CSA may have suicidal ideations when they feel guilty, which remains consistent with previous studies to some extent. A systematic review reported that there exists an association between state guilt and suicidal behavior [d = 0.29 (95% CI:0.06, 0.51), I²=67%] [77]. The earlier analysis of suicide also found that feeling guilty seems to be an aggravating underlying emotional factor among men for attempting suicide, which acts as a punishment for those feelings of guilt [78]. Once disclosing their experience of CSA, men may meet negative responses in the form of stigma and stereotypes (i.e., blaming, wagging, ignoring) [79], which could increase the severity of guilt symptoms to reflect perceived inward interpersonal failure and negative empathic recognition [80]. Guilt is one of the mixed emotions in the affective system to trigger suicidal ideation and behavior [81]. Furthermore, female youth with CSA are more likely to generate suicide-related thoughts or attempts when they experience uncontrollable worry. A study among patients with mood disorders broadly revealed that patients with severe anxiety symptoms (including worry symptoms) showed a strong correlation to suicidal thoughts and behaviors compared to their counterparts with mild symptoms (P < 0.001) [82]. Women appear to have a greater tendency toward emotional disorders, which could incur more suicide-related outcomes across the life course [83]. Existing research indicates that worry is one of the core repetitive negative thinking (RNT) patterns in psychiatric disorders connected to suicide [84], which links to suicidal ideation and attempts (i.e., feeling hopeless, burdensome) [85]. And worry is crucial in suicidality among those suffering from co-occurring depression and anxiety symptoms [86]. Thus, women who experienced CSA may incur repetitive and elevated levels of worry [87] and may generate more frequent suicide attempts [88].

We exposed a strong correlation between being afraid and being able to relax within the anxiety and depression network structure among CSA male survivors, which was reported in previous network analyses as well [89, 90]. Worries or fears of unsafety and recurrent sexual abuse may hinder the ability to relax [91]. In line with previous network structures of anxiety and depression [37, 57], restlessness is highly correlated to female CSA survivors’ inability to relax. This may be attributed to the fact that women, after CSA, frequently encounter sleep disturbances causing restlessness [92]. Sustained studies have tried to explain the sex difference between anxiety and depression according to neurons and their mechanisms. From the transcriptomic perspective, there were sex-specific transcriptional profiles in cortical regions and limbic systems among patients with major depressive symptoms [93]. And the male brain’s microglia could be activated at lower numbers, where males have much more dense spines than their female counterparts once they have entered a severe-depressive mood [94]. Research on arginine vasopressin and oxytocin illustrated that these matters could activate the same neuro system in both sexes but produce anxiety only for males [30].

This study held the superiority of large-scale sample size and utilized a network approach to disclose symptomatic characteristics of anxiety and depression among survivors of CSA. Central symptoms could predict changes to other symptoms, and bridge symptoms could be clinically transdiagnostic to differences between the two symptoms [95]; hence, targeted interventions toward these symptoms could be imposed to relieve anxiety and depressive disorders for CSA survivors. Despite this, several limitations were noted in this study. First, this cross-sectional study was conducted within one month so that there may include a portion of participants confronted with acute psychological disorders rather than long-term chronic ones. Thus, longitudinal studies are required to investigate further the comparison between chronic and acute psychological issues, and to explore more causal relationships between symptoms. Second, the research team did not have the resources to confirm a clinical diagnosis from a psychiatrist and relied on self-report, thus, this study may include some participants who met the clinical standard for anxiety and depression. Further studies based on samples with clinical diagnosis should be conducted in the future. Third, the direction of edges failed to be identified, requiring further research to understand the networks of those with CSA better. Fourth, the network approach has the inherent flaw of limited replicability across samples [96]. Meanwhile, scales of subjective reports on anxiety and depression may be met with report bias and memorial errors. Finally, the results based on a unique population of CSA survivors should be carefully explored before being able to generalize to other populations.

To conclude, this study uncovered central and bridge symptoms (control worry, sad mood, energy) between anxiety and depression network structure among CSA survivors. Meanwhile, sex differences between the two network models were also presented, particularly in the case of suicidal ideations and suicide attempts. Further, longitudinal studies are required focusing on CSA populations and their psychiatric symptoms. Advisable targeted interventions and clinical measurements could be created using this evidence base to relieve comorbid anxiety and depression symptoms for CSA populations.

### Electronic supplementary material

Below is the link to the electronic supplementary material.

Supplementary Material 1

## Data Availability

The datasets used and analyzed during the current study are available from the corresponding author upon reasonable request.
