# Supplementary material for: Anxiety and Depression Symptoms among Youth Survivors of Childhood Sexual Abuse: A Network Analysis
Source: BMC Psychol. 2023 Sep 16;11:278. doi: 10.1186/s40359-023-01275-3 (PMC10504753; doi:10.1186/s40359-023-01275-3)
Supplement: Supplementary file 1 — Supplementary Material 1 [file 40359_2023_1275_MOESM1_ESM.docx]

**Supplementary Materials**

Figure S1. The adjusted network structure of anxiety and depression after controlling for sex, family type, current annual income, and only-child status.

Figure S2. Strength, Betweenness, and Closeness in the network of anxiety and depression.

Figure S3. Bridge Strength, Bridge Betweenness, Bridge Closeness in the network of anxiety and depression.

Figure S4. Bootstrapped confidence intervals of edge weights.

Figure S5. The stability of centrality and bridge centrality indices using case-dropping bootstrap.

Figure S6. Estimation of edge weight difference by bootstrapped difference test.

Figure S7. The male network structure of anxiety and depression.

Figure S8. The male network structure of bridge symptoms of anxiety and depression.

Figure S9. The female network structure of anxiety and depression.

Figure S10. The female network structure of bridge symptoms of anxiety and depression.

Figure S11. The stability of centrality and bridge centrality indices using case-dropping bootstrap for male and female networks.

Figure S12. Bootstrapped confidence intervals of edge weights for male and female networks.

Figure S13. Estimation of male and female edge weight difference by bootstrapped difference test.

Table S1. Male VS Female: Results of independent t-test.

Figure S1. The adjusted network structure of anxiety and depression after controlling for sex, family type, current annual income, and only-child status.


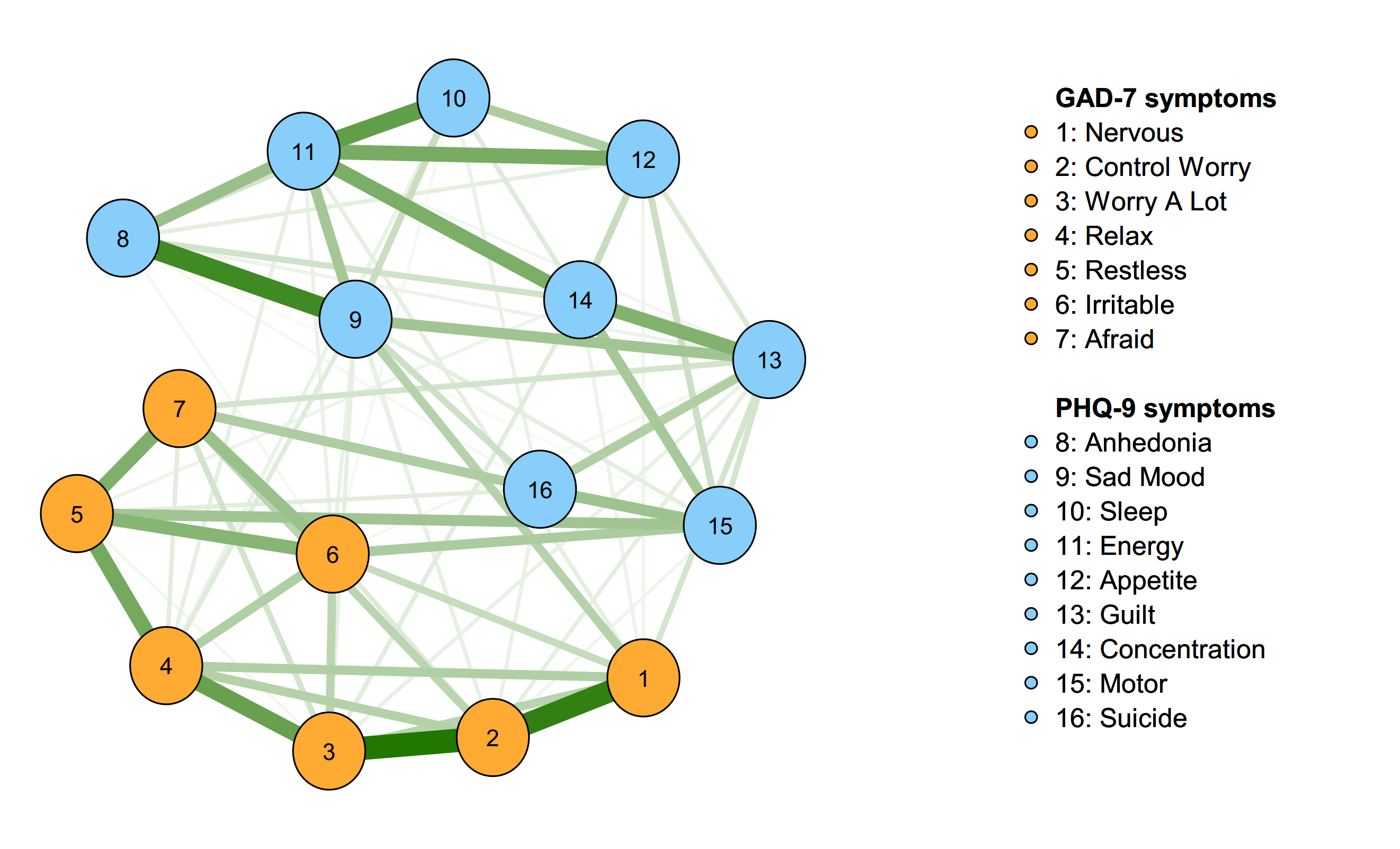


Figure S2. Strength, Betweenness, and Closeness in the network of anxiety and depression.


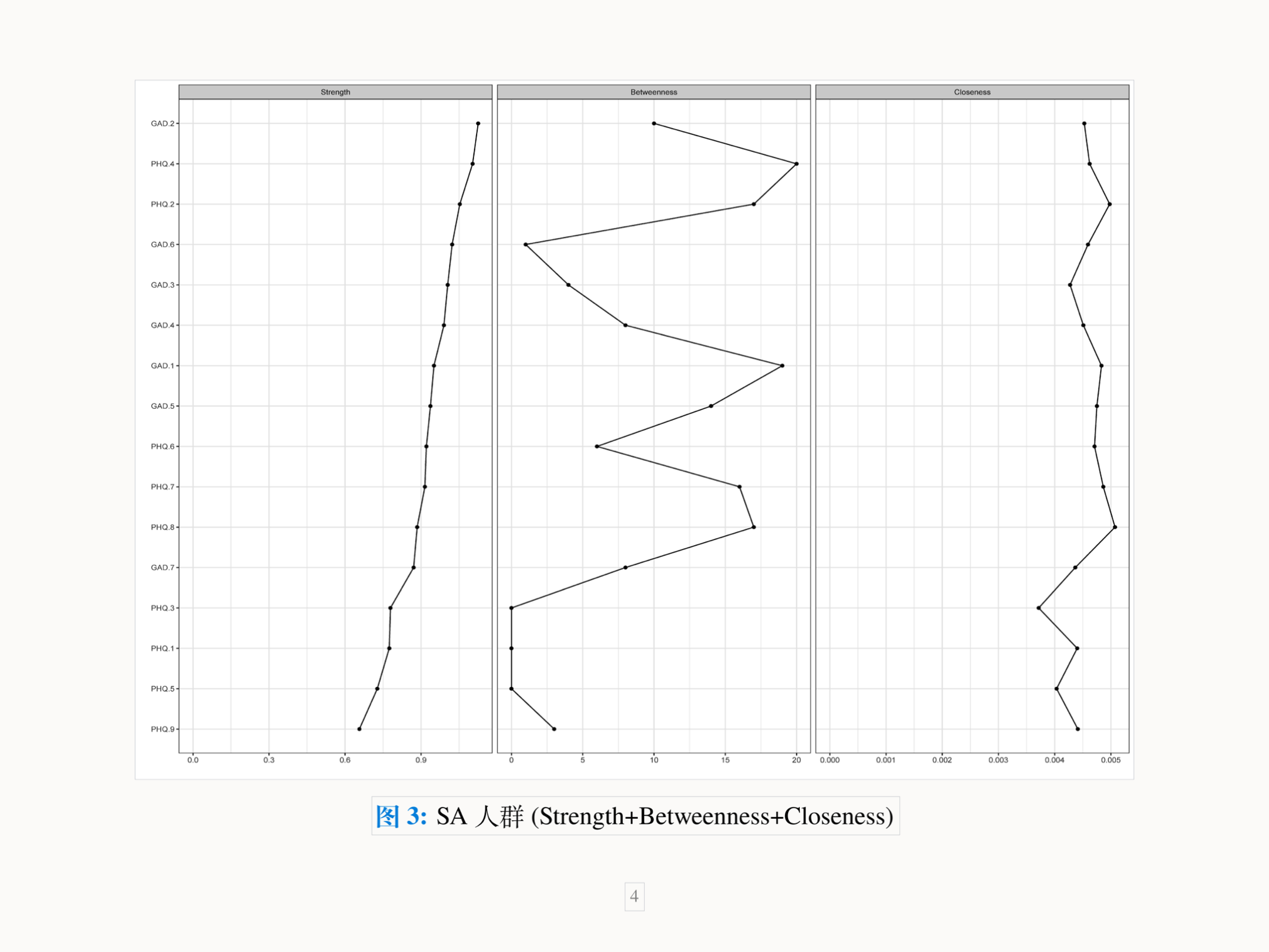


Figure S3. Bridge Strength, Bridge Betweenness, Bridge Closeness in the network of anxiety and depression.


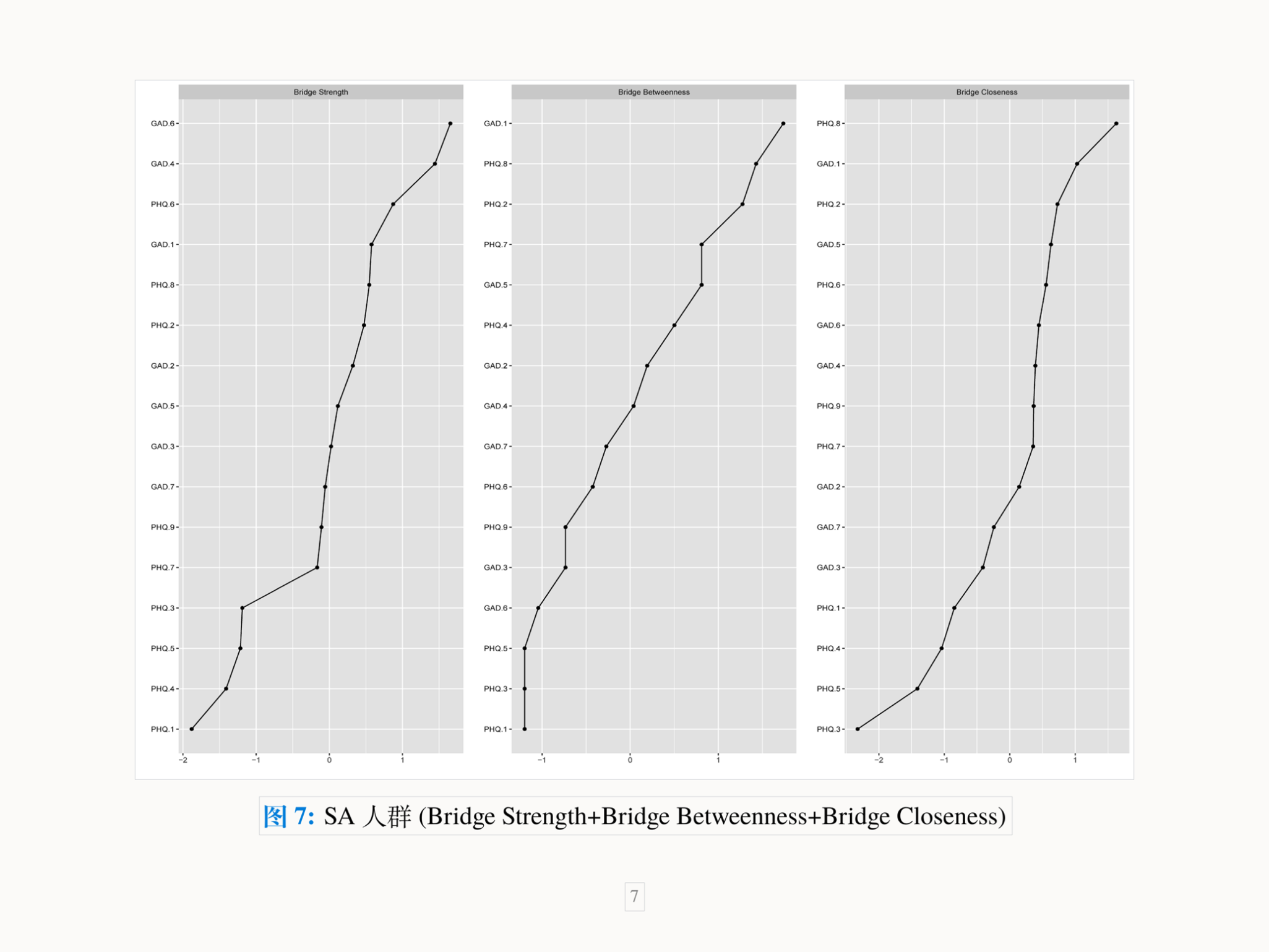


Figure S4. Bootstrapped confidence intervals of edge weights.


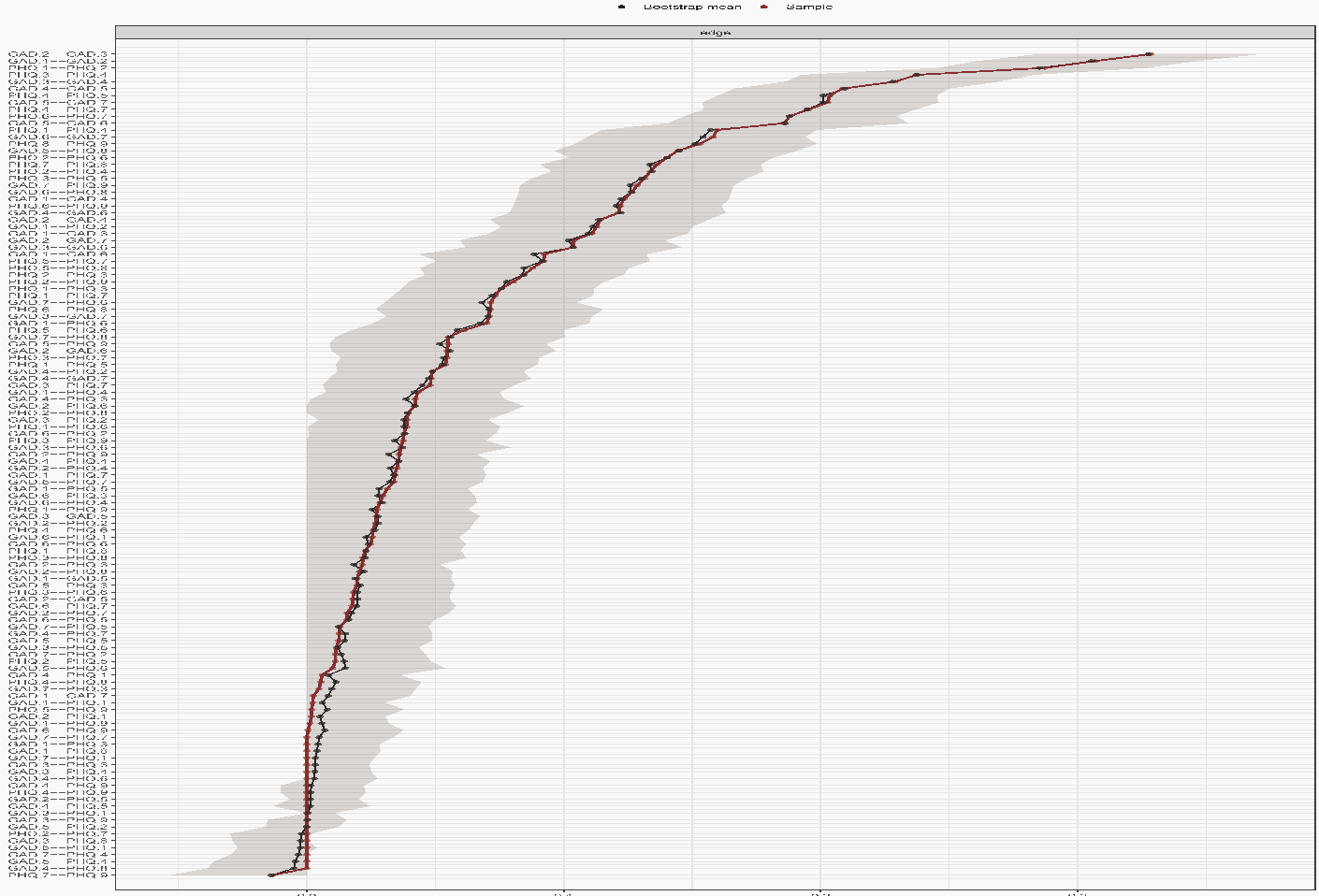


Figure S5. The stability of centrality and bridge centrality indices using case-dropping bootstrap.


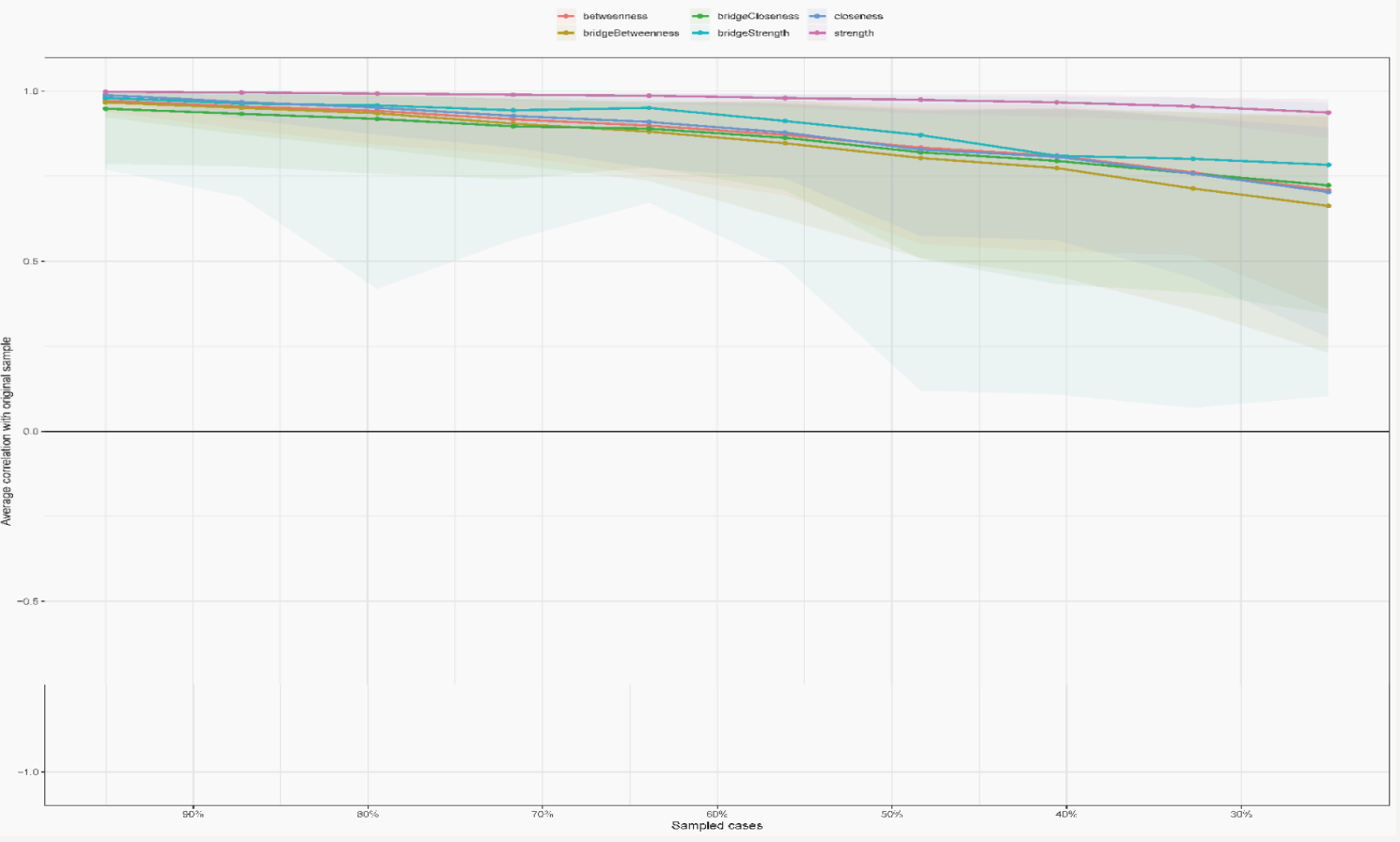


Figure S6. Estimation of edge weight difference by bootstrapped difference test.


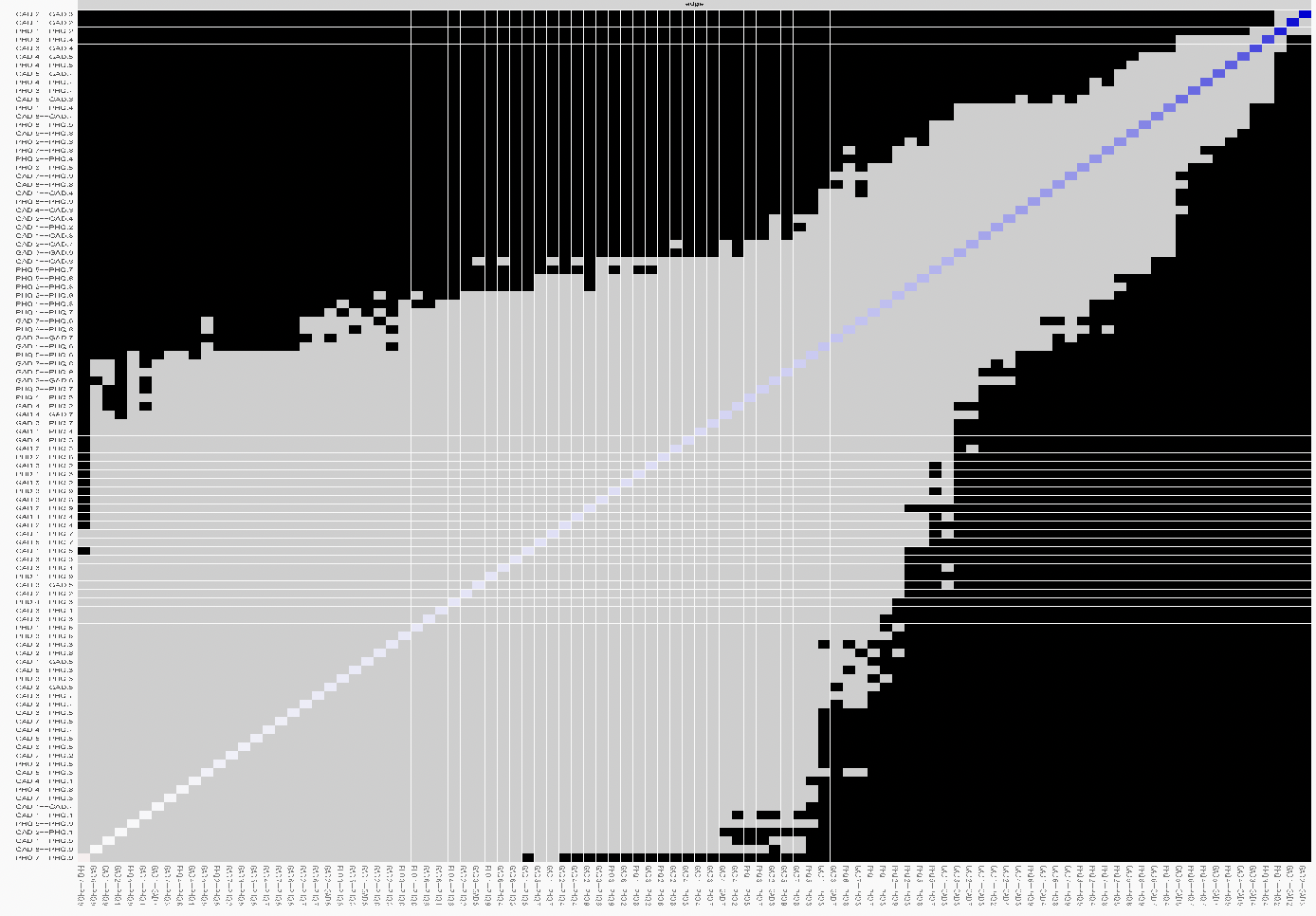


Figure S7. The male network structure of anxiety and depression.
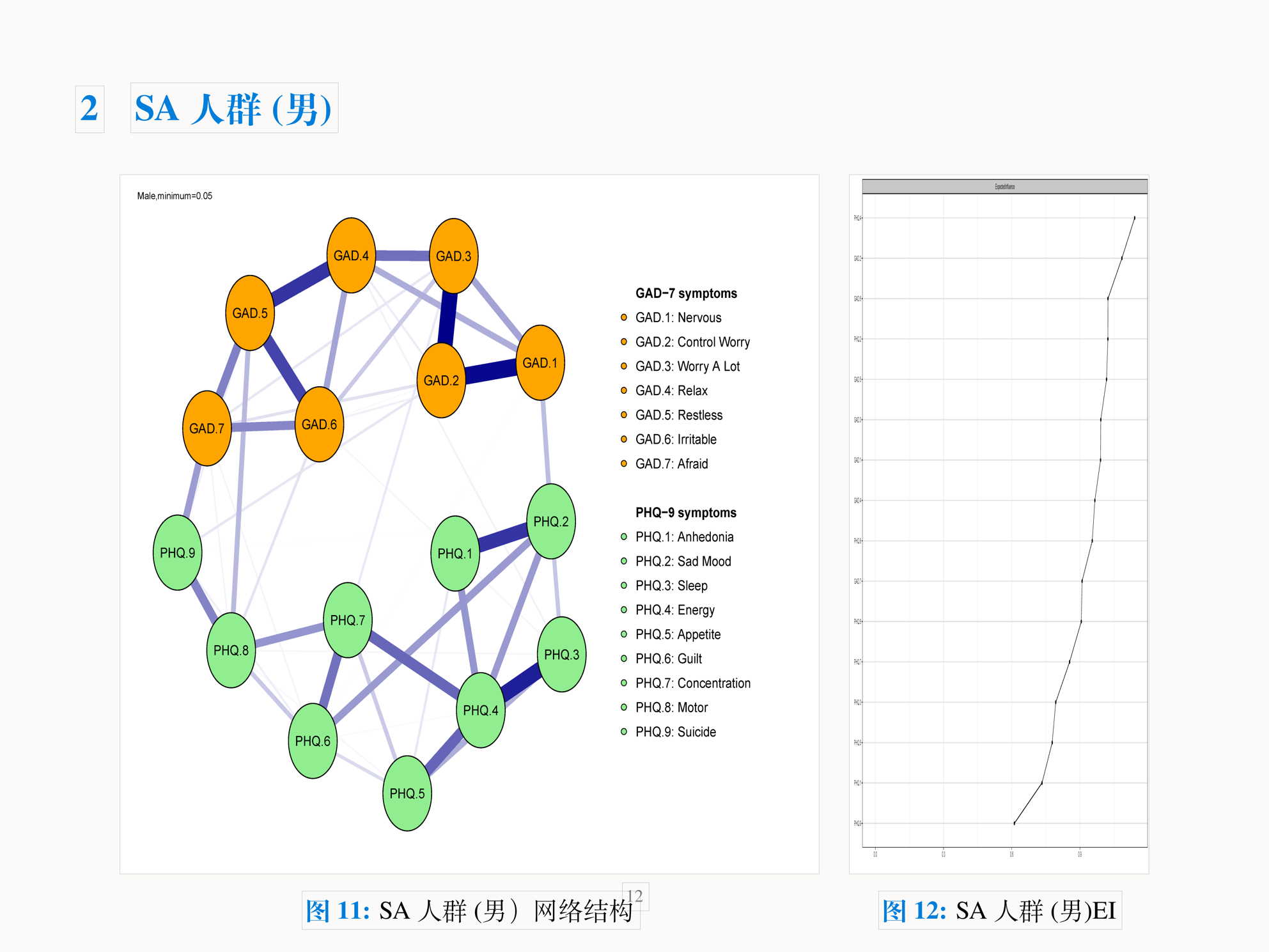


Figure S8. The male network structure of bridge symptoms of anxiety and depression.
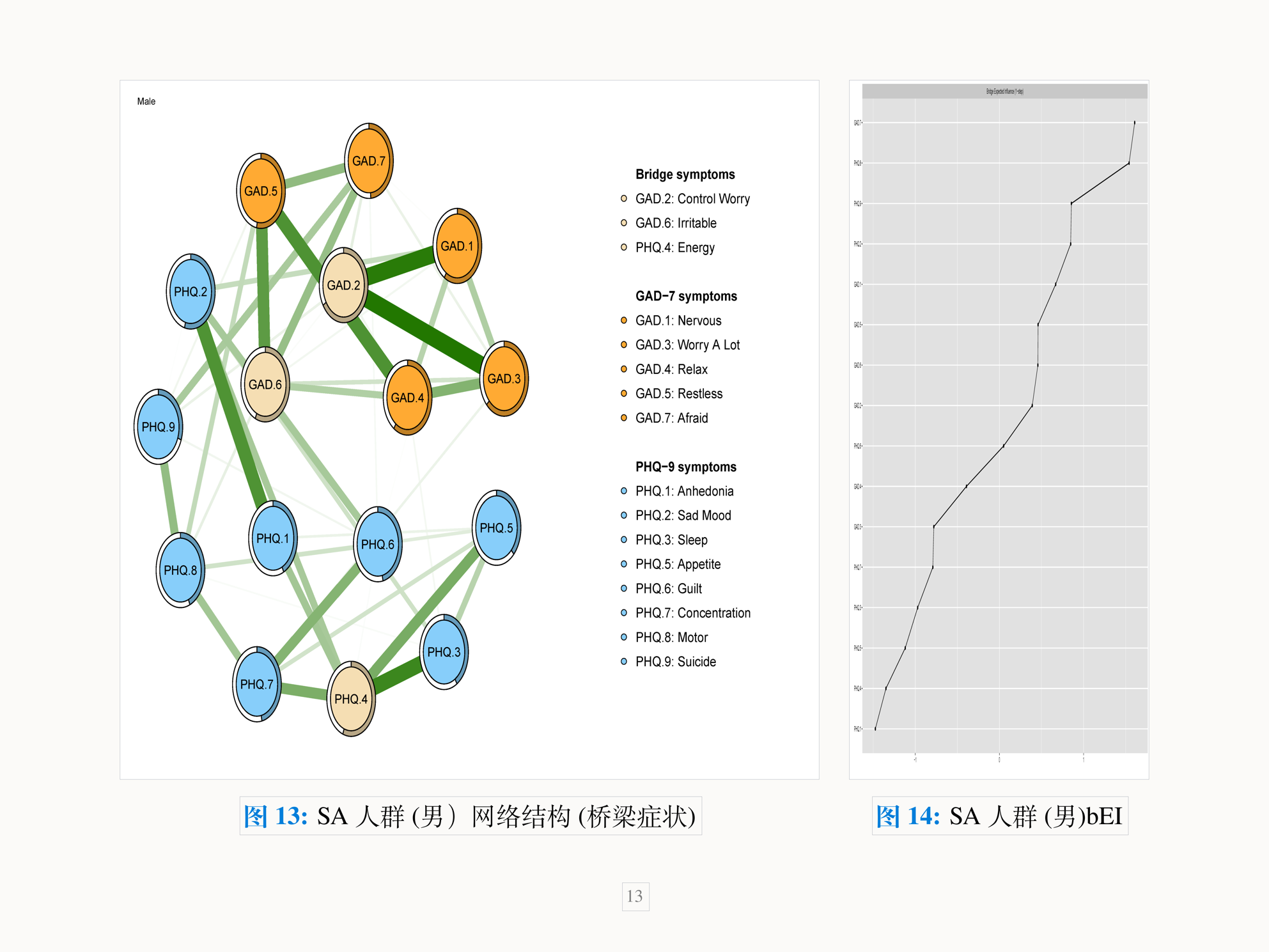


Figure S9. The female network structure of anxiety and depression.
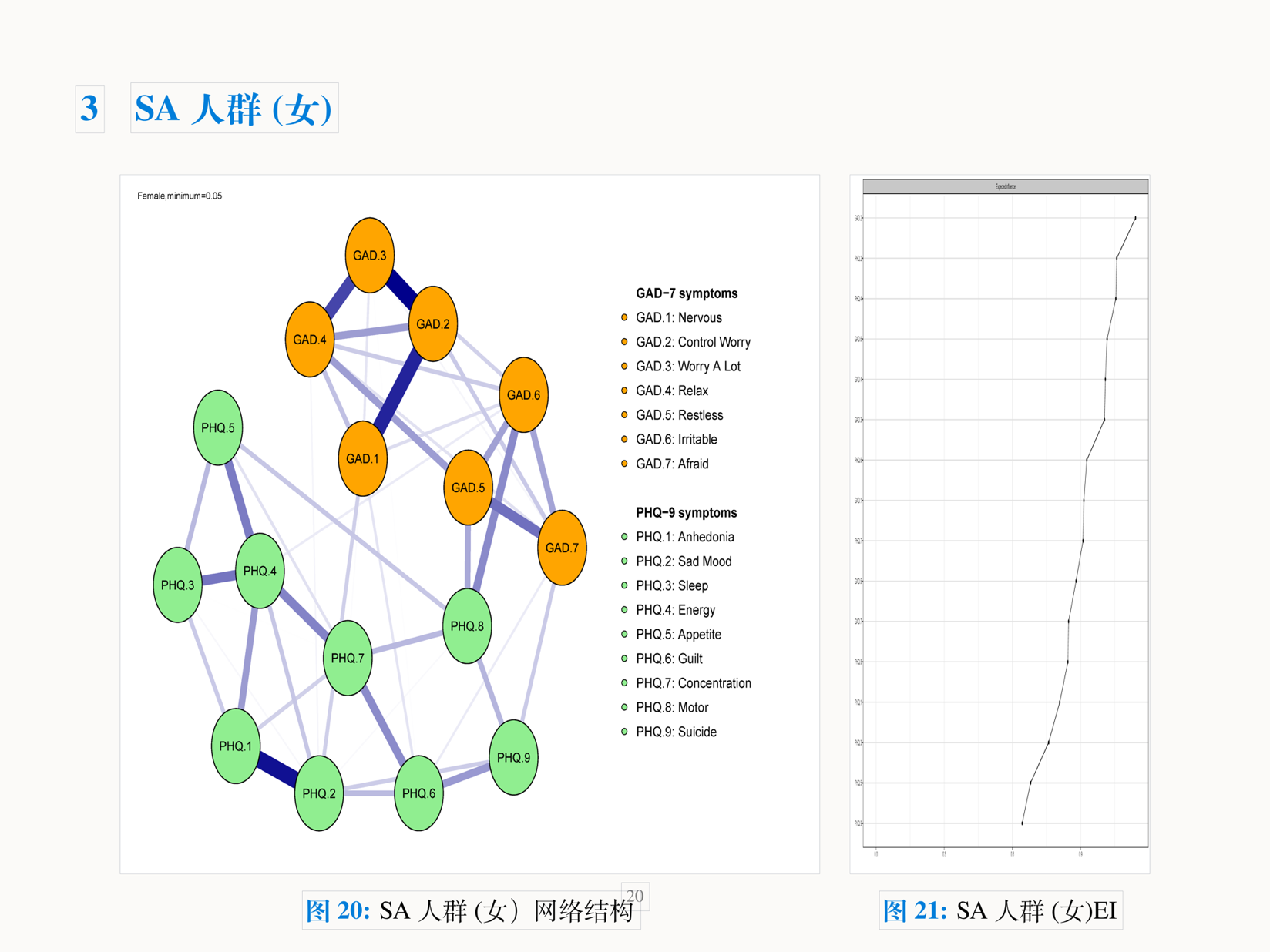


Figure S10. The female network structure of bridge symptoms of anxiety and depression.
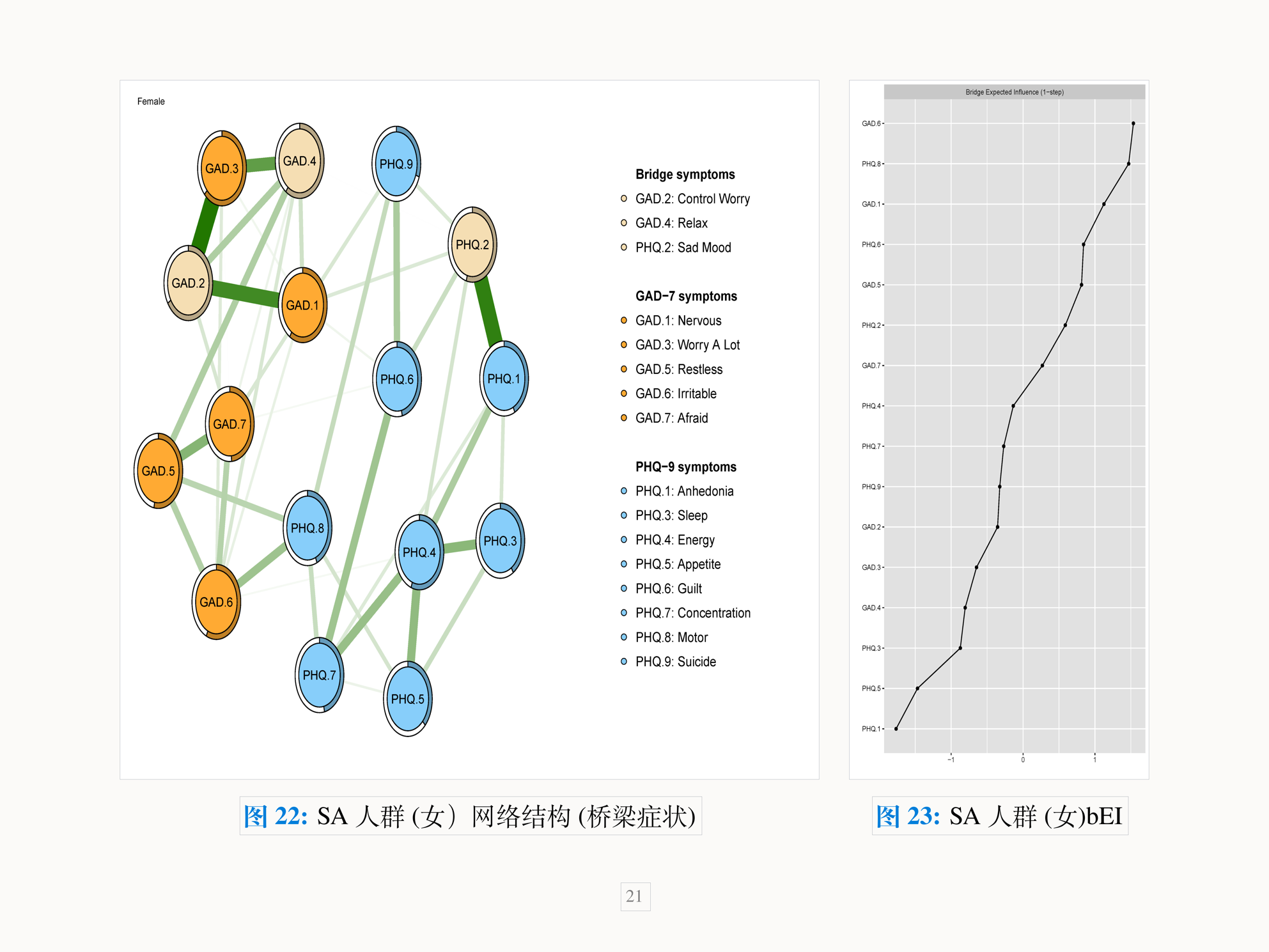


Figure S11. The stability of centrality and bridge centrality indices using case-dropping bootstrap for male and female networks.


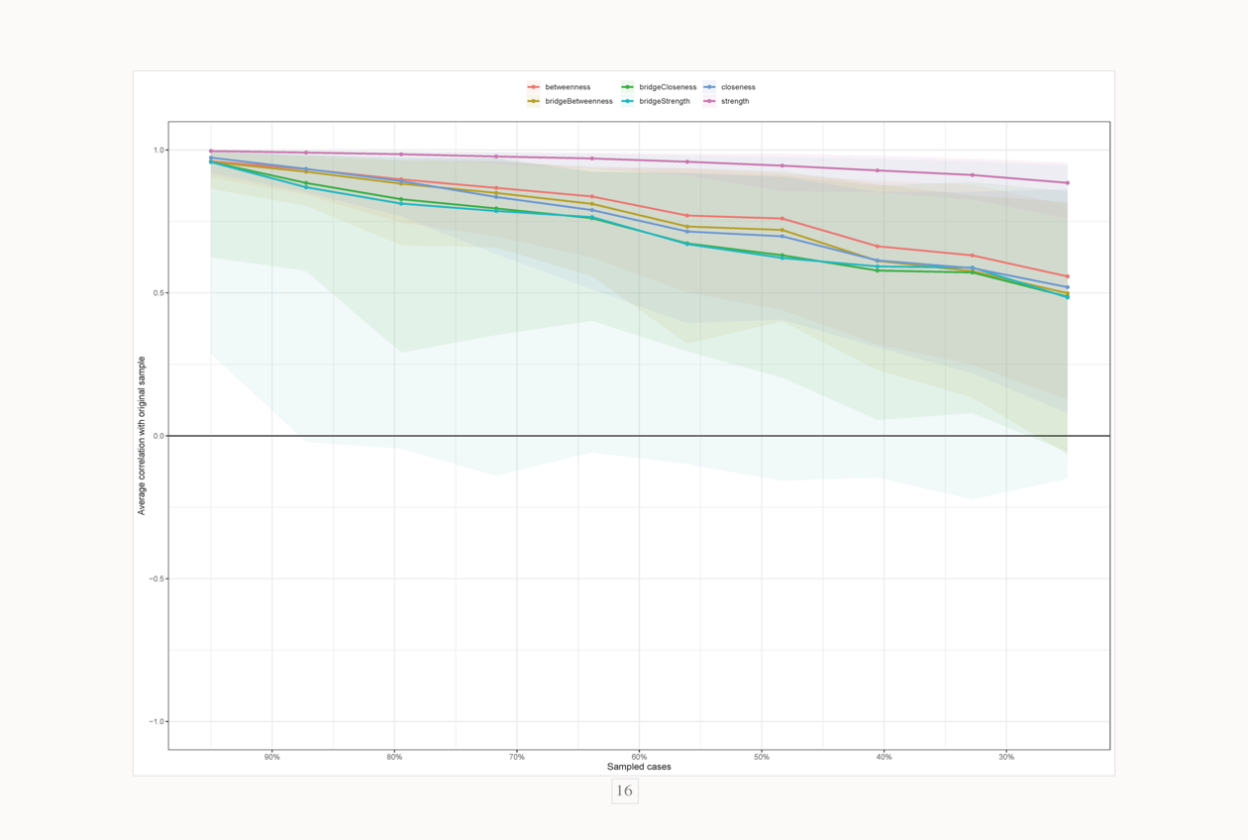

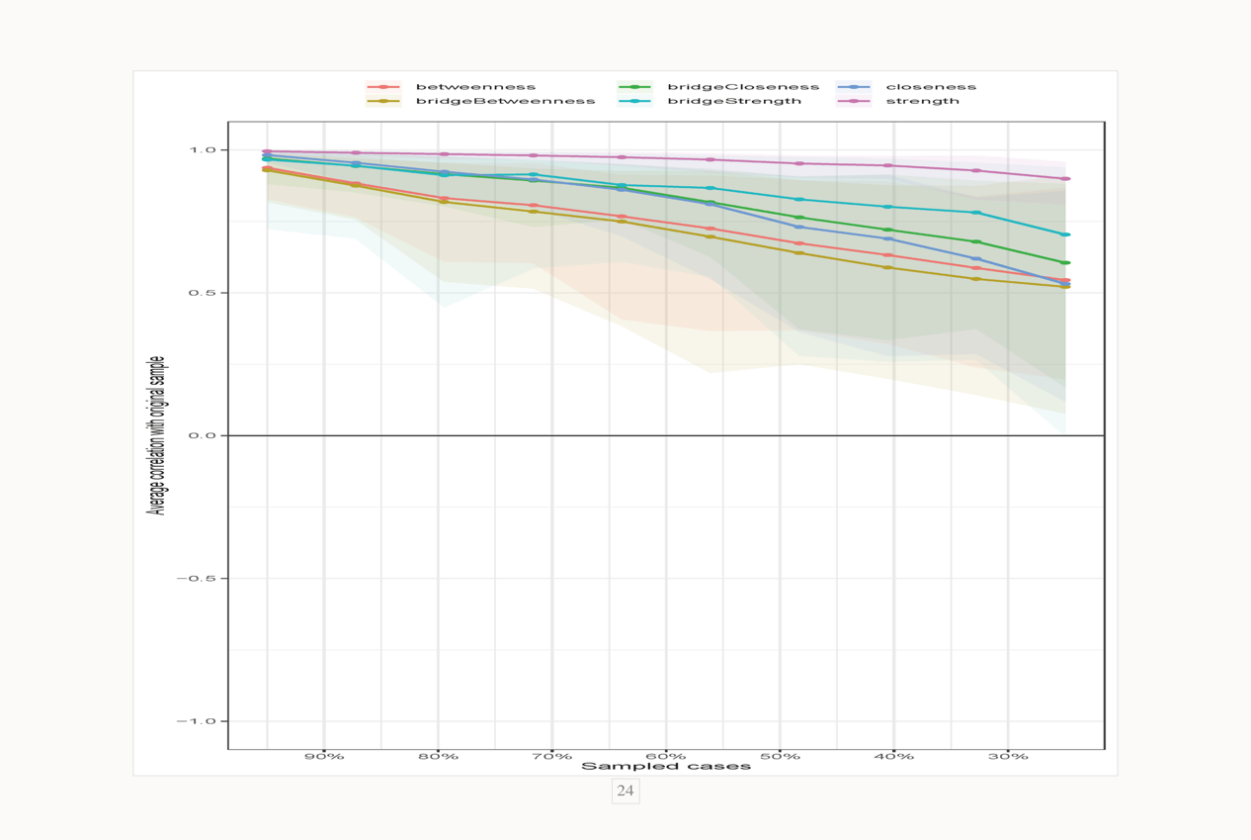


Figure S12. Bootstrapped confidence intervals of edge weights for male and female networks.


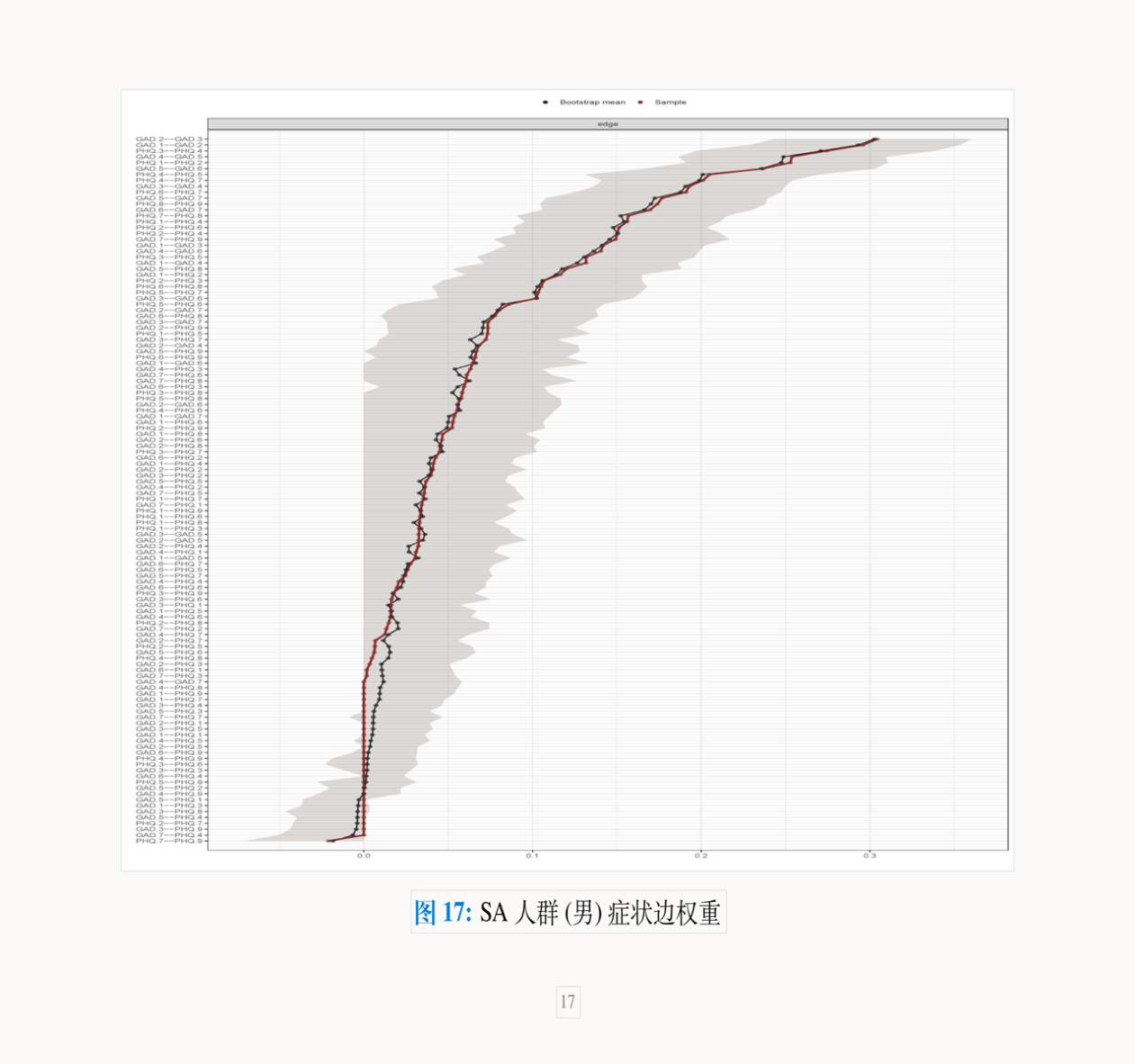

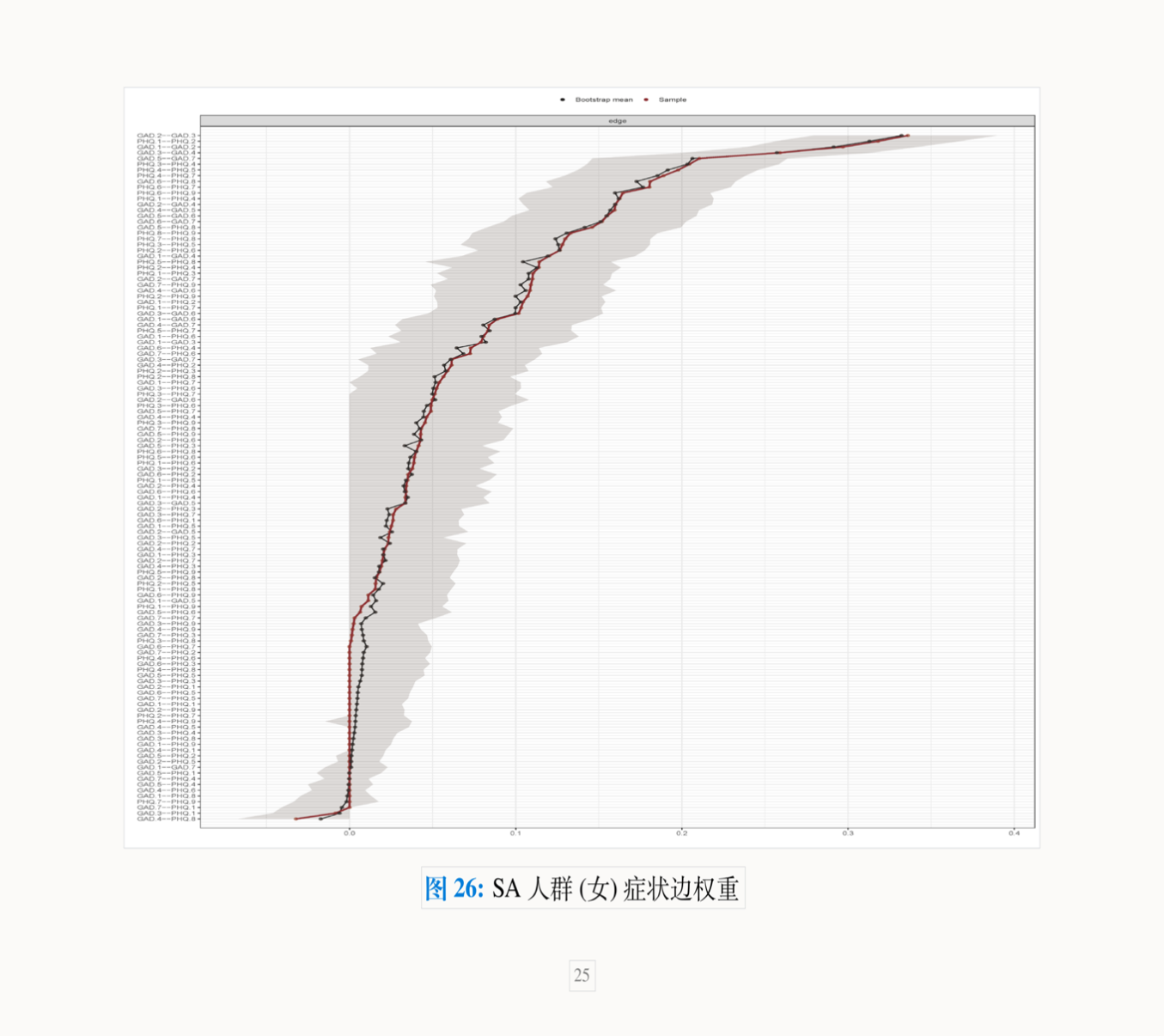


Figure S13. Estimation of male and female edge weight difference by bootstrapped difference test.


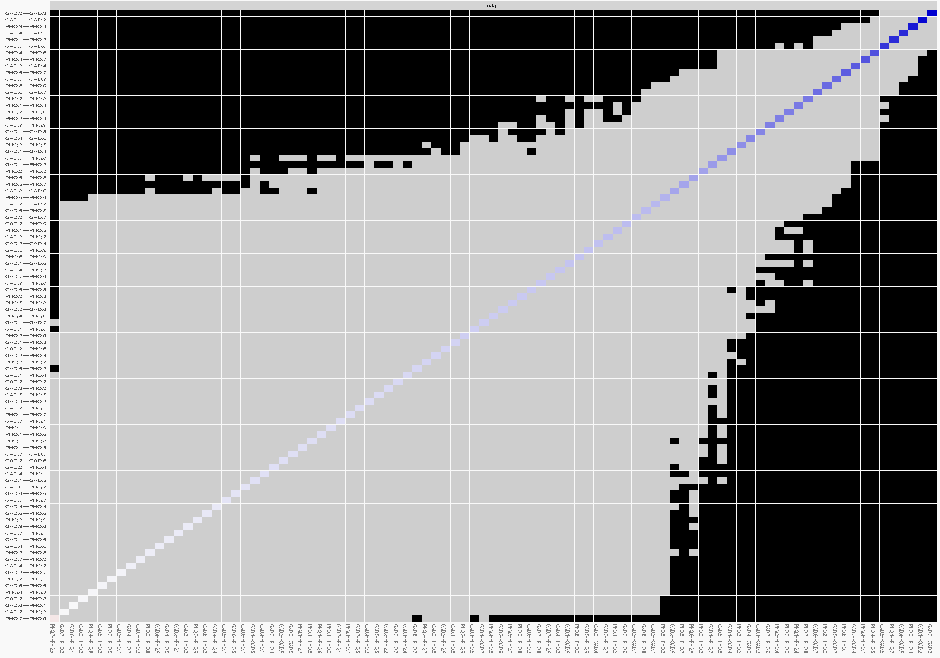

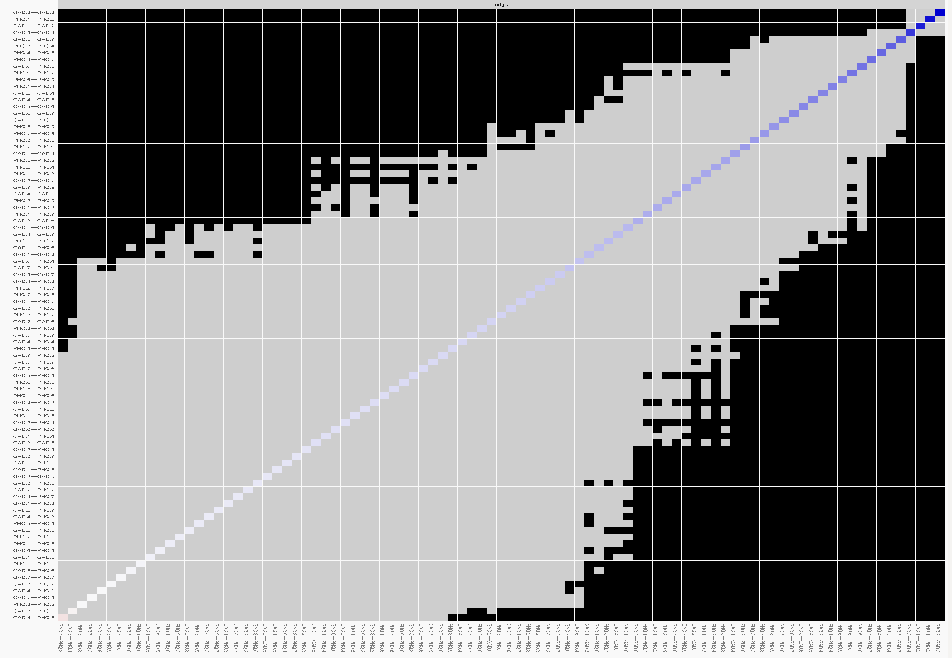


|  |  |  |  |  |  |  |  |  |  |
| --- | --- | --- | --- | --- | --- | --- | --- | --- | --- |
| **Table S1** | Male VS. Female: Results of independent t-test | | Scale | Symptoms | MD | SD | T value | P value | 95 % CI |
|  |  |  | GAD-7 | Nervous | -0.25 | 0.02 | -10.12 | **<0.001** | (-0.30, -0.20) |
|  |  |  |  | Control Worry | -0.23 | 0.03 | -8.56 | **<0.001** | (-0.28, -0.18) |
|  |  |  |  | Worry A Lot | -0.2 | 0.02 | -7.32 | **<0.001** | (-0.26, -0.15) |
|  |  |  |  | Relax | -0.11 | 0.03 | -4.03 | 0.019 | (-0.17, -0.06) |
|  |  |  |  | Restless | -0.06 | 0.04 | -1.39 | 0.088 | (-0.14, 0.02) |
|  |  |  |  | Irritable | -0.19 | 0.03 | -7.29 | 0.025 | (-0.24, -0.14) |
|  |  |  |  | Afraid | -0.05 | 0.02 | -1.98 | 0.091 | (-0.10, 0.00) |
|  |  |  | PHQ-9 | Anhedonia | -0.13 | 0.02 | -5.39 | **<0.001** | (-0.18, -0.08) |
|  |  |  |  | Sad Mood | -0.12 | 0.02 | -5.20 | **<0.001** | (-0.17, -0.07) |
|  |  |  |  | Sleep | -0.16 | 0.03 | -5.62 | 0.035 | (-0.22, -0,11) |
|  |  |  |  | Energy | -0.18 | 0.03 | -6.57 | 0.169 | (-0.23, -0.12) |
|  |  |  |  | Appetite | -0.19 | 0.03 | -6.80 | 0.158 | (-0.25, -0.14) |
| MD: Mean difference; SD: Standard error; CI: Confidence interval; GAD-7, the seven-item Generalized Anxiety Disorders Scale; PHQ-9, the nine-item Patient Health Questionnaire | | |  | Guilt | -0.10 | 0.03 | -3.83 | 0.025 | (-0.15, -0.05) |
|  |  |  |  | Concentration | -0.17 | 0.03 | -5.95 | 0.025 | (-0.23, -0.11) |
|  |  |  |  | Motor | 0.01 | 0.02 | 0.36 | 0.623 | (-0.04, 0.06) |
|  |  |  |  | Suicide | -0.06 | 0.02 | -3.27 | **<0.001** | (-0.10, -0.03) |
